# Supplementary material for: Multiple exciton generation boosting over 100% quantum efficiency photoelectrochemical photodetection
Source: Nat Commun. 2025 Jun 6;16:5275. doi: 10.1038/s41467-025-60420-1 (PMC12144185; doi:10.1038/s41467-025-60420-1)
Supplement: Supplementary file 1 — Supplementary Information [file 41467_2025_60420_MOESM1_ESM.pdf]

Supporting information

Multiple exciton generation boosting over 100% quantum efficiency photoelectrochemical photodetection

Junjun Xue [1], Xu Wang [1], Guanyu Xu [1], Xinya Tao [1], Tongdao Pan [1], Zhouyu Chen [2], Qing Cai [3], Pengfei Shao[3], Guofeng Yang [4*], Zengli Huang [5], Ting Zhi [1], Ke Wang [3], Bin Liu [3], Dunjun Chen [3*], Rong Zhang [3], and Jin Wang [1*]*

[1] GaN-X Laboratory, College of Electronic and Optical Engineering & College of Flexible Electronics (Future Technology), Nanjing University of Posts and Telecommunications, Nanjing, 210023, China.

[2] Portland Institute of NJUPT, Nanjing University of Posts and Telecommunications, Nanjing, 210023, China.

[3] Key Laboratory of Advanced Photonic and Electronic Materials, School of Electronic Science and Engineering, Nanjing University, Nanjing, 210093, China.

[4] School of Science, Jiangnan University, Wuxi, 214122, China.

[5] Suzhou Laboratory, Suzhou, 215123, China.

e-mail: pfshao@nju.edu.cn; gfyang@jiangnan.edu.cn; djchen@nju.edu.cn; jin@njupt.edu.cn

Section 1. Supplementary Figures

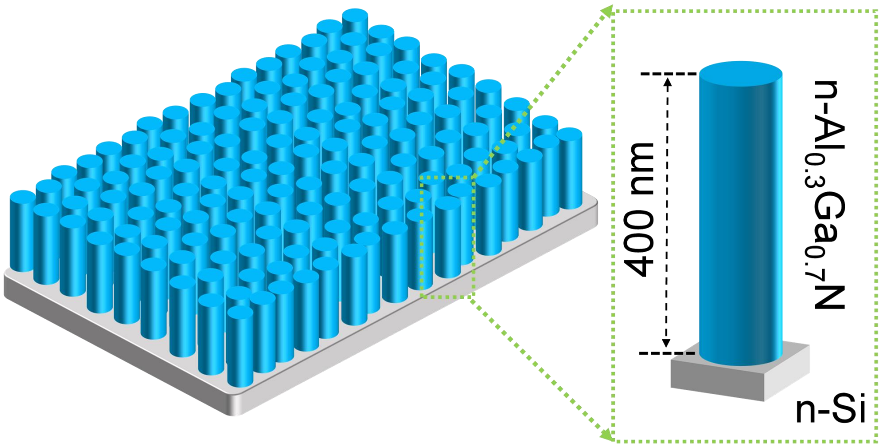

Supplementary Figure 1. The structure of as-grown n-AlGaN nanowires on n-Si substrate. The photoelectrode, adopted for this work, is composed of Si-doped n-Al0.3Ga0.7N nanowires (~400 nm long), vertically grown on n-Si (111) substrate.

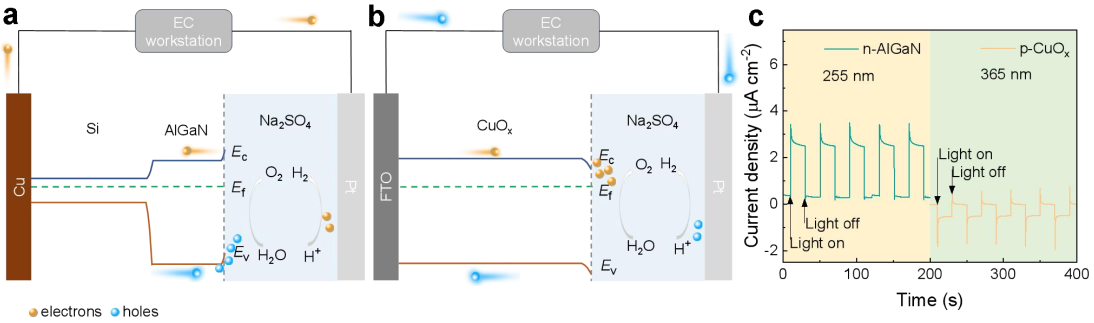

Supplementary Figure 2. Working mechanism diagrams of N-type and P-type semiconductors. The energy band structures of the n-AlGaN/electrolyte (a) and p-CuO_x/electrolyte (b) hetero-interface. (c), The current signal of n-AlGaN and p-CuO_x PEC PDs. When bare n-type AlGaN contacts an electrolyte solution, the reduction potential difference leads to a built-in electric field, causing the surface energy band of AlGaN to bend upward at the interface. When UV light irradiate the AlGaN photoelectrode, photogenerated charge carriers are excitated and then separated at the AlGaN/Na_2SO_4 interface[1–3]. The photogenerated holes migrate toward the electrolyte, reach the semiconductor/electrolyte interface, and participate in the PEC oxidation reaction ($2H_2O + 4h^+ \rightarrow O_2 + 4H^+$). The corresponding photogenerated electrons enter the counter electrode circuit through an external connection and participate in the PEC reduction reaction ($2H^+ + 2e^- \rightarrow H_2$). Thus, photogenerated electrons can be captured by electrochemical workstation according to the external circuit, resulting in a positive photocurrent. Here, the PEC device can function as a photodetector under self-powered conditions, with the assist of built-in electric field at the semiconductor/electrolyte interface driving the separation of electrons and holes. Similarly, when the bare p-type CuO_x contacts with the electrolyte solution, the surface energy bands of CuO_x bend downward at the interface. Upon irradiation with ultraviolet light, the photogenerated electrons migrate towards the electrolyte and participate in the reduction reaction ($2H^+ + 2e^- \rightarrow H_2$). Correspondingly, the photogenerated holes enter the counter electrode through the external circuit and take part in the oxidation reaction ($2H_2O + 4h^+ \rightarrow O_2 + 4H^+$). The migration directions of both electrons and holes are opposite to those of the previously mentioned n-type AlGaN, so a negative photocurrent is generated.

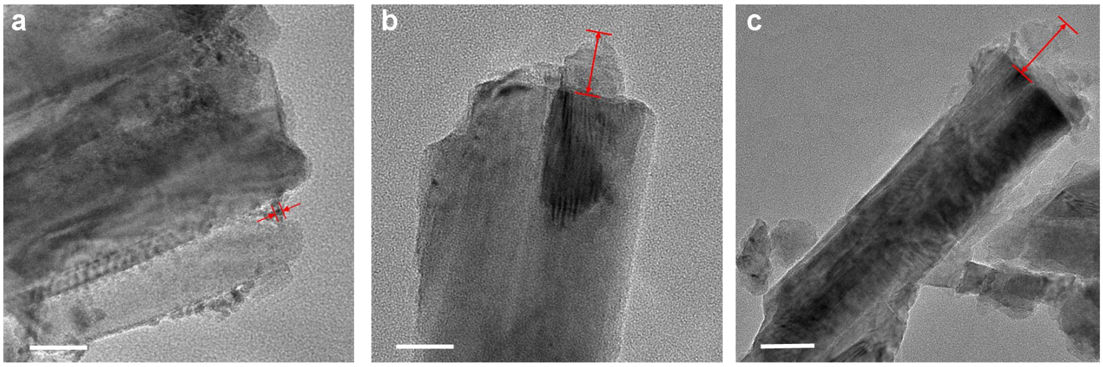

Supplementary Figure 3. The TEM images of AlGaN/CuO_x samples with different CBD time. The thickness of CuO_x on AlGaN for CBD-10 (a; scale bar, 20 nm), CBD-30 (b; scale bar, 20 nm) and CBD-50 (c; scale bar, 30 nm) are ~3, ~20 and ~50 nm, respectively.

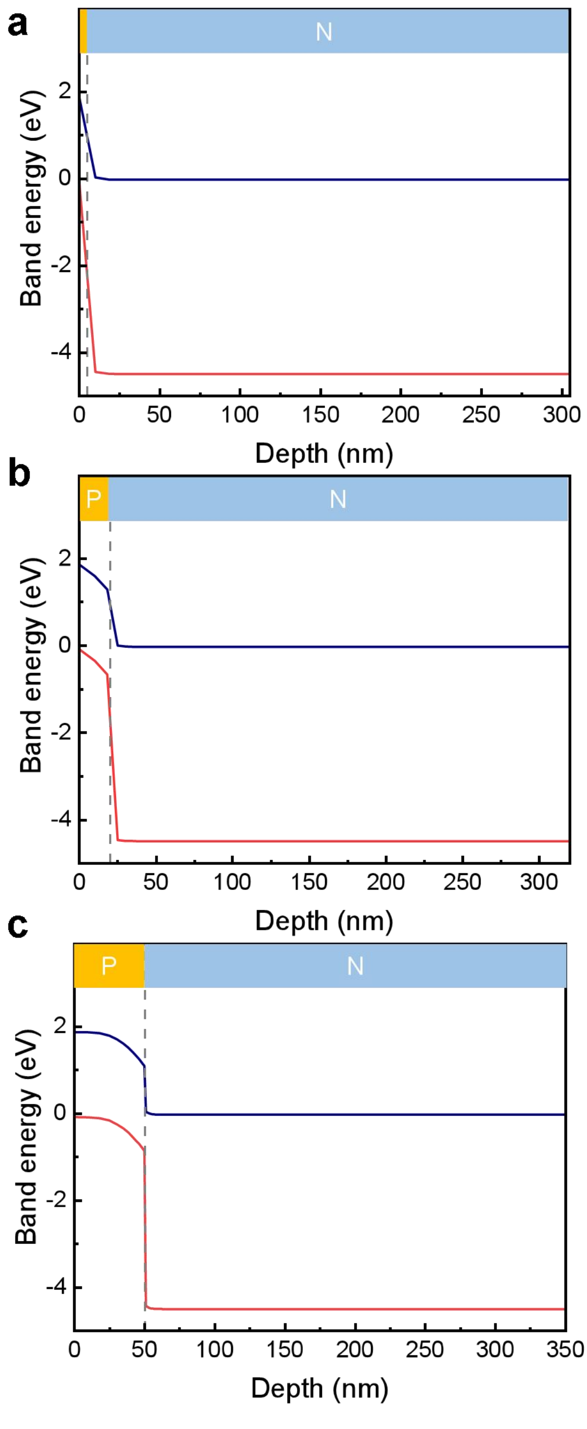

Supplementary Figure 4. The AlGaN/CuO_x samples band structure of different CBD time simulated by Silvaco TCAD. The tilt energy band of CuO_x suggests the cladding oxides layer are totally depleted by the n-AlGaN nanowire for CBD-10 (a) and CBD-30 (b). Nevertheless, when the thick of CuO_x layer increase to 50 nm for CBD-50 (c), the energy band of CuO_x become flattened at the far end of the junction and the the downward band bending could be established at the interface of un-depleted CuO_x/electrolyte.

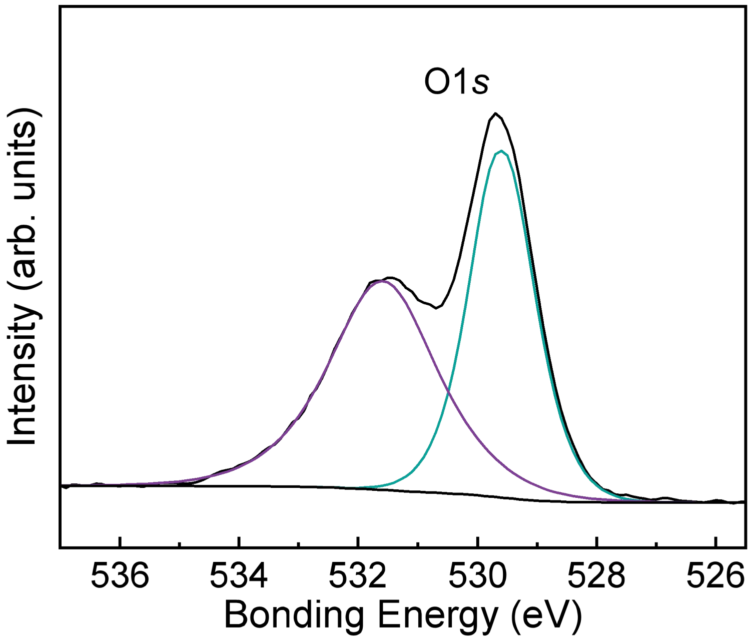

Supplementary Figure 5. The XPS spectrum of O1*s*. The peak at 529.5 eV corresponds to the metal-oxygen bonding, and the peak locating at 531.6 eV could be attributed to surface hydroxide in the XPS spectrum of O1*s*.

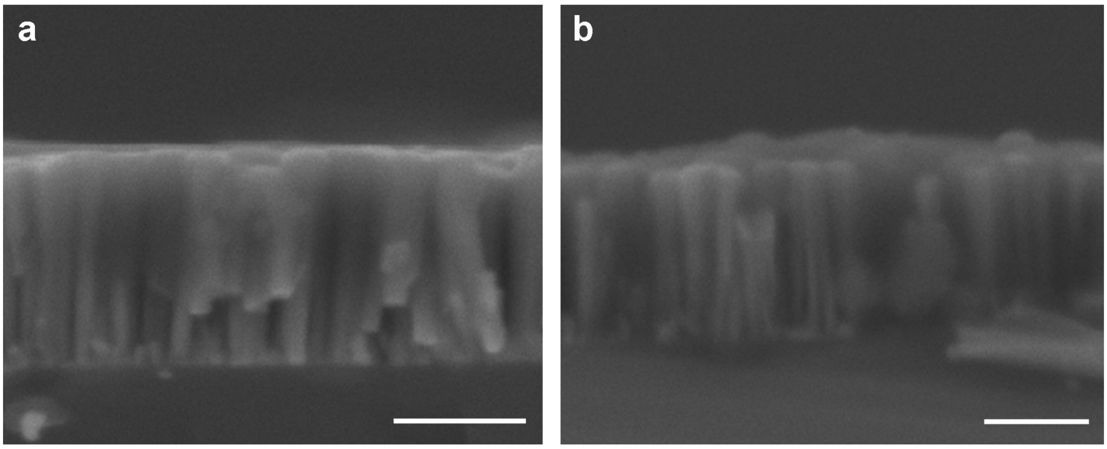

Supplementary Figure 6. SEM cross-sectional image of nanowires. (a), AlGaN (scale bar, 250 nm), (b), AlGaN/CuO_x (scale bar, 250 nm). The CuO_x layer resides atop the AlGaN NWs (~400 nm long).

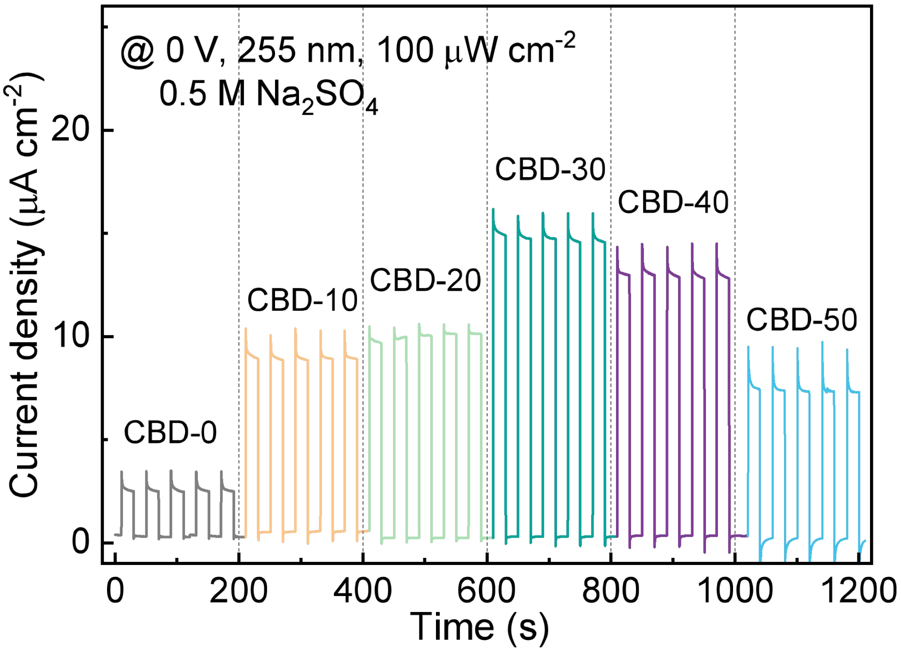

Supplementary Figure 7. I-t curves of AlGaN/CuO$_x$ PEC PDs at different CBD time. These depicts photoelectrochemical measurements of the original AlGaN nanowires and AlGaN/CuO$_x$ samples with different impregnation times under UV irradiation at a light intensity of 100 μW cm^{-2}. The photocurrent density results show that the current density first increased and then decreased with increasing CuO$_x$ modification time, showing that the 30-minute CuO$_x$ deposition corresponds the highest photoresponse. The photo-current density of sample CBD-30 was recorded as 17.39 μA cm^{-2} and is 6.1 times higher than that of the original sample (CBD-0). With increased deposition time, the amount of CuO$_x$ on the AlGaN nanowire surface increases, completing the construction of AlGaN/CuO$_x$ heterostructure.

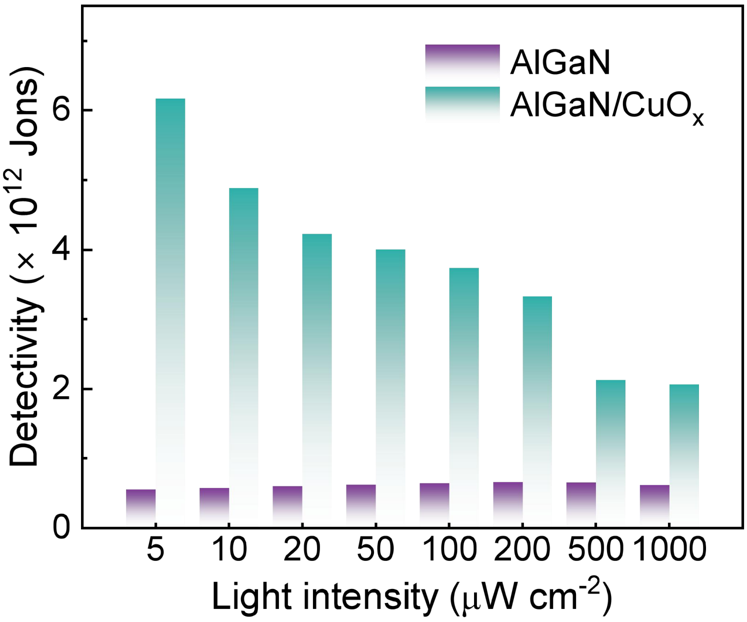

Supplementary Figure 8. The specific detectivity of bare AlGaN and AlGaN/CuO_x PEC PDs under 255 nm illumination at different light power. The AlGaN and AlGaN/CuO_x PEC PDs exhibit remarkable D^* value of 5.56×10^{11} and 6.17×10^{12} Jones at 5 μW cm^{-2}, respectively, revealing the ability of AlGaN/CuO_x PEC-type PD to detect weak light.

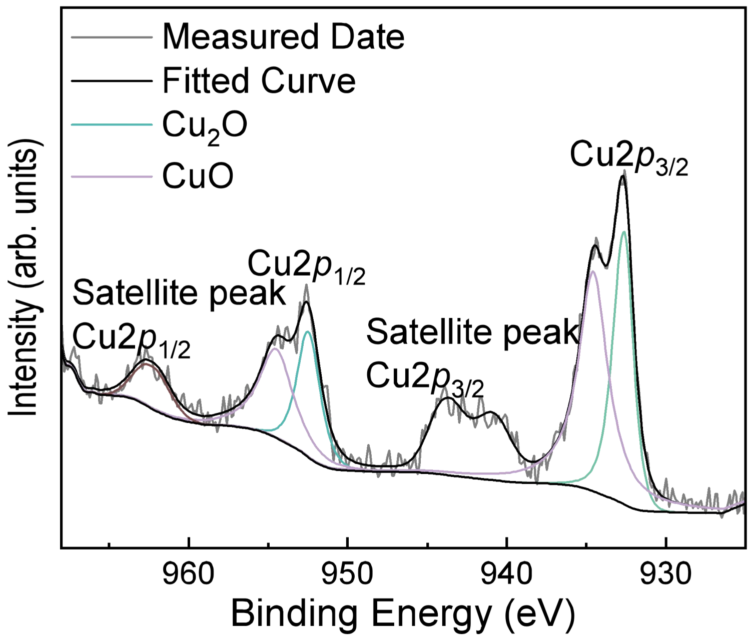

Supplementary Figure 9. The XPS characterization of Cu_xO grown on a quartz substrate. The CuO_x sample deposited on a quartz substrate (see Methods) was tested for XPS with the Cu2*p* characteristic peak consistent with that of the AlGaN/CuO_x sample.

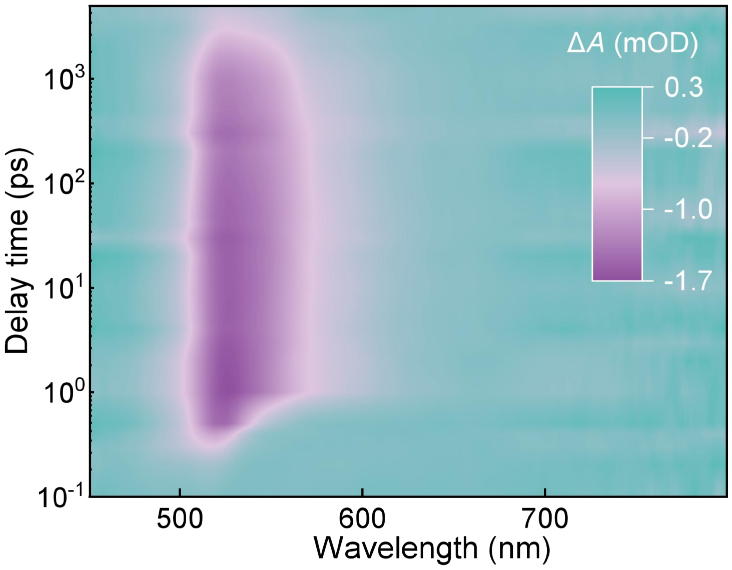

Supplementary Figure 10. The 3D color image of time-resolved femtosecond transient absorption spectra excited by a 10 nJ cm^{-2} flux density at 300 nm. The figure shows the time-resolved transient absorption spectra measured at a pump energy of 4.13 eV, a ground-state bleaching (GSB) peak appears near 520 nm under the excitation pulse.

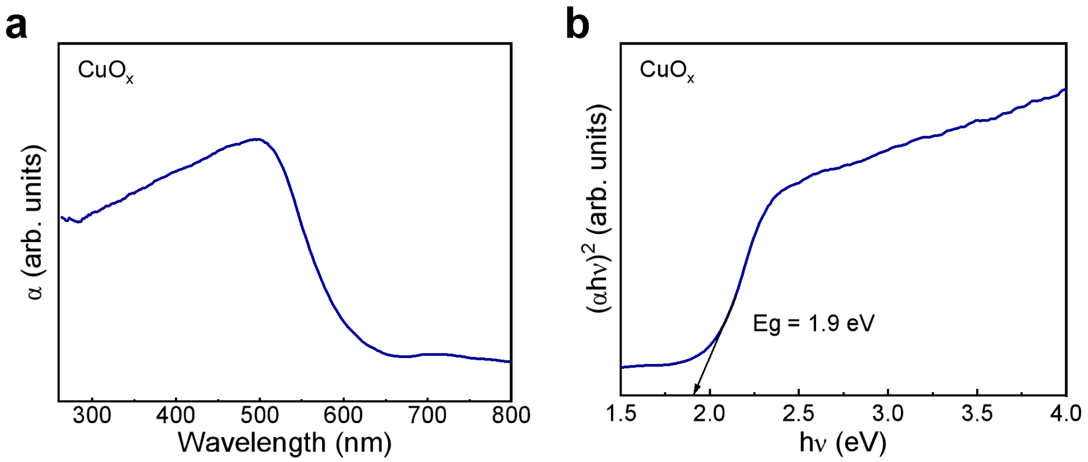

Supplementary Figure 11. UV-vis absorption spectra of CuO$_x$. (a), CuO$_x$ absorption spectrum in the range of 250-800nm. (b), plot of $(\alpha h\nu)^2$ versus hν for the E_g of CuO$_x$.

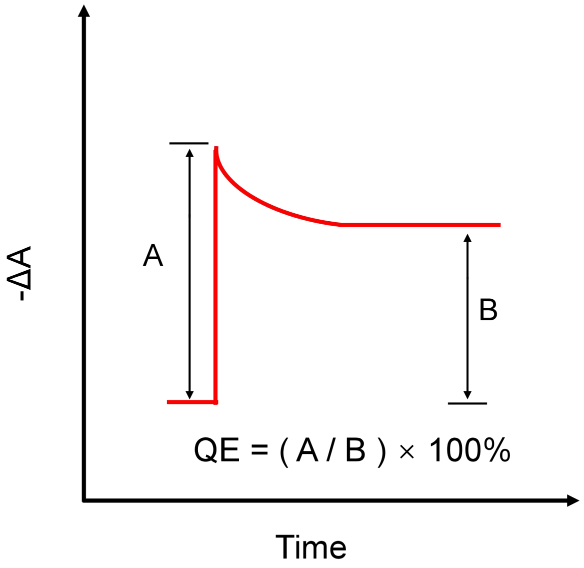

Supplementary Figure 12. The approximate calculation of MEG QE. The TA signal rapidly decays from the initial peak to a steady value. The ratio of the peak signal strength A to the platform signal strength B is proportional to the average number of excitons generated in each stimulated nanosemiconductor.

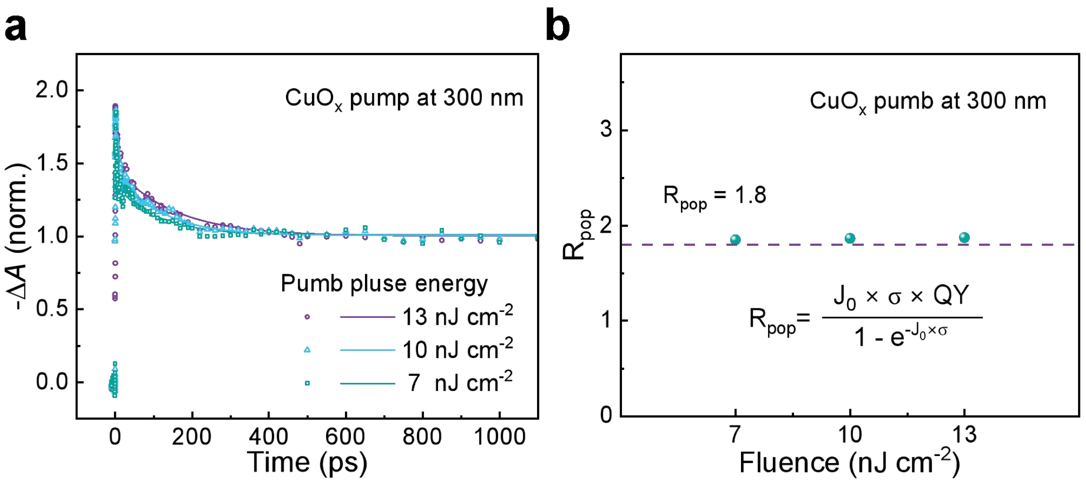

Supplementary Figure 13. **The ground state bleaching dynamics of CuO_x under different pump fluence.** (a) GSB dynamics and R_{pop} under different pump fluence at 300 nm. The TA spectroscopy was conducted for probing the dynamics of ground state bleaching (GSB). The average number of excitons generated per absorbed photon (QY) can be determined by following equation:

$$R_{pop} = \frac{J_0 \times \sigma \times QY}{1 - e^{-J_0 \times \sigma}} \quad (1)$$

Where R_{pop} is the exciton population ratio of excitons generated directly following excitation and long delays when Auger recombination is complete, J_0 is the photo fluence of the pump pulse, σ is the absorption cross section at the pump wavelength, and at low fluence where $(1 - e^{-J_0 \times \sigma}) \rightarrow J_0\sigma$, then $R_{pop} \rightarrow$ QY. The GSB dynamics of CuO_x at different fluence generated by 300 nm light are showen in Supplementary Fig. 13a. The R_{pop} at 7, 10 and 13 nJ cm^{-2} are almost constant. R_{pop} reaches fluence independent regime, indicating the absence of multiphoton absorption (see Supplementary Fig. 13b).

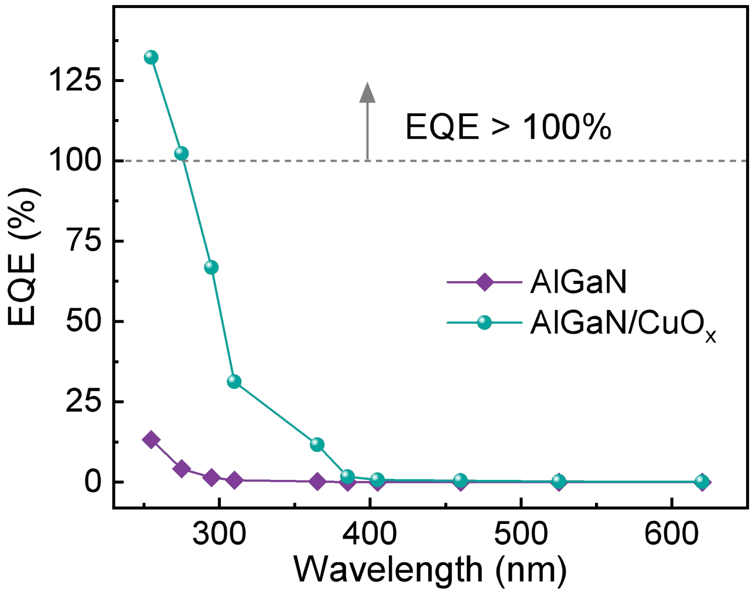

Supplementary Figure 14. EQE as a function of wavelength for the bare AlGaN and AlGaN/CuO_x PEC PD at 0 V bias. It is clearly indicated that EQE of AlGaN/CuO_x PD exceeds 100% irradiated by ultraviolet light below 300 nm.

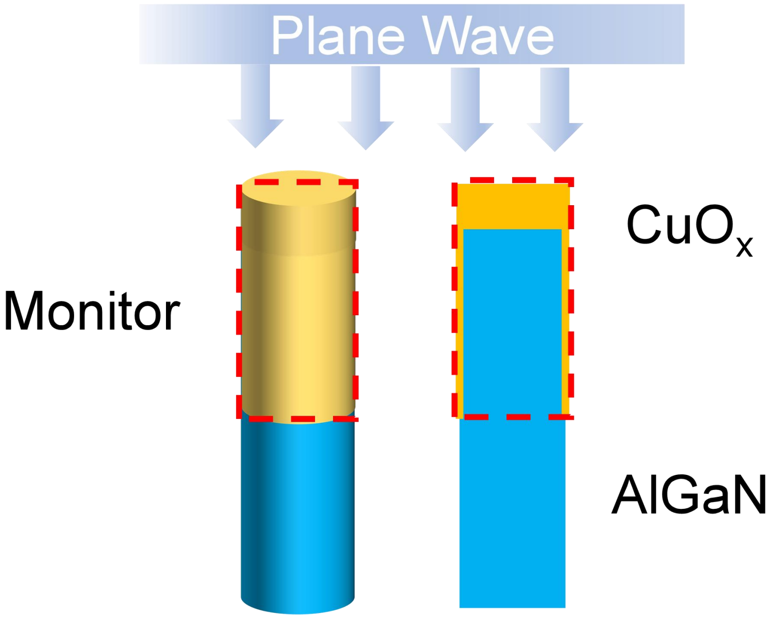

Supplementary Figure 15. Schematic diagram of the numerical model of AlGaN/CuO_x nanowires heterojunction. The AlGaN/CuO_x core-shell structure is constructed for finite-difference time-domain (FDTD) simulation, where the AlGaN nanowire is 400 nm long, the CuO_x top layer is 20 nm thick, the CuO_x shell is 3 nm, and the light source is a plane wave.

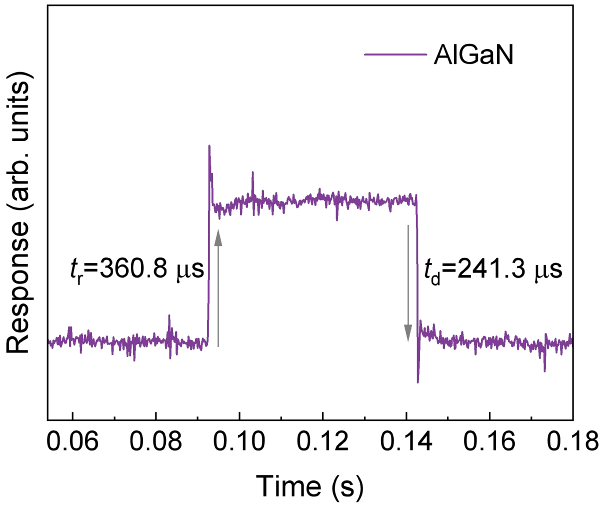

Supplementary Figure 16. The rise and decay times for bare-AlGaN PEC PD. The response speeds of the bare AlGaN nanowires was 360.8 μs and 241.3 μs, respectively.

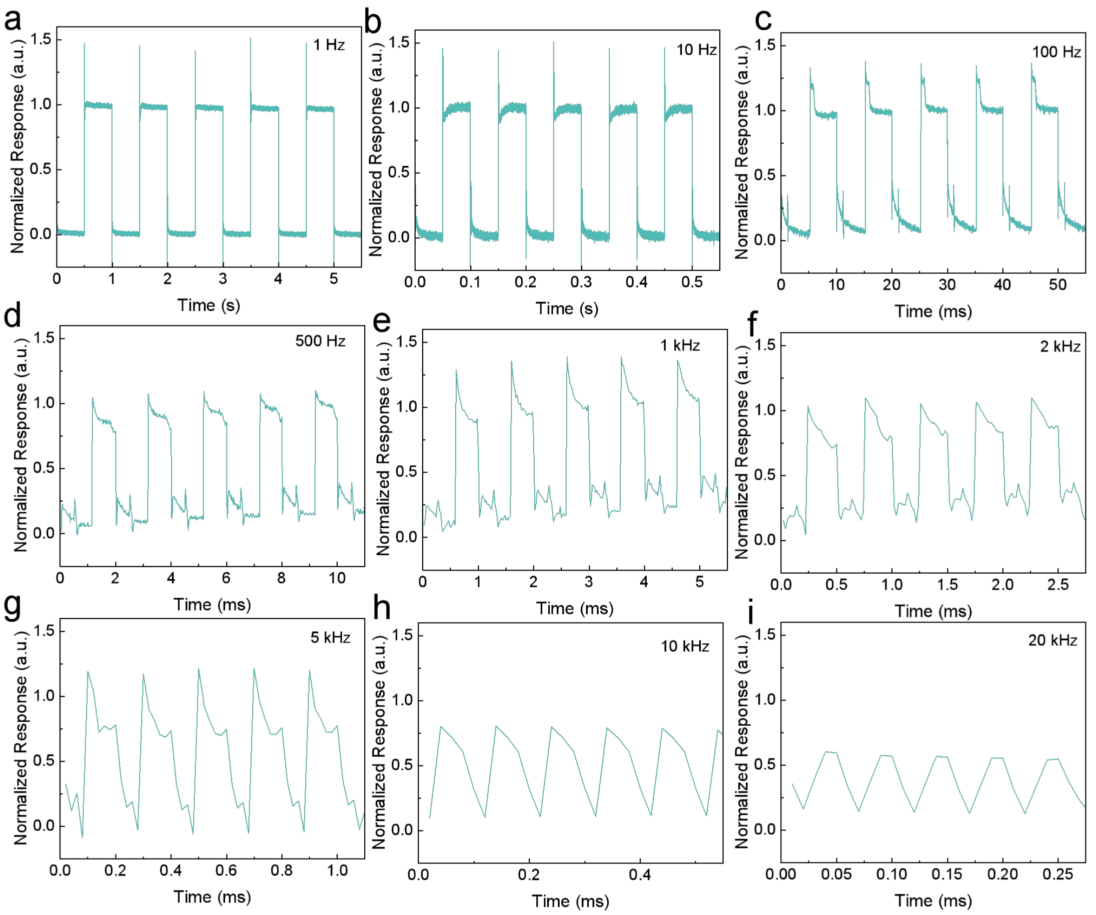

Supplementary Figure 17. Normalized response curves at various transmitting frequencies. (a) 1 Hz, (b) 10 Hz, (c) 100 Hz, (d) 500 Hz, (e) 1 kHz, (f) 2 kHz, (g) 5 kHz, (h) 10 kHz, (i) 20 kHz. It can be observed that the output waveform remains stable as a square wave with clear rising/falling edges up to 1 kHz, yet when the frequency increases to 10 kHz, the output waveform transitions from a square wave to a triangular wave, probably because the PD reaches its response-speed limit and lacks sufficient time to stabilize at high frequencies.

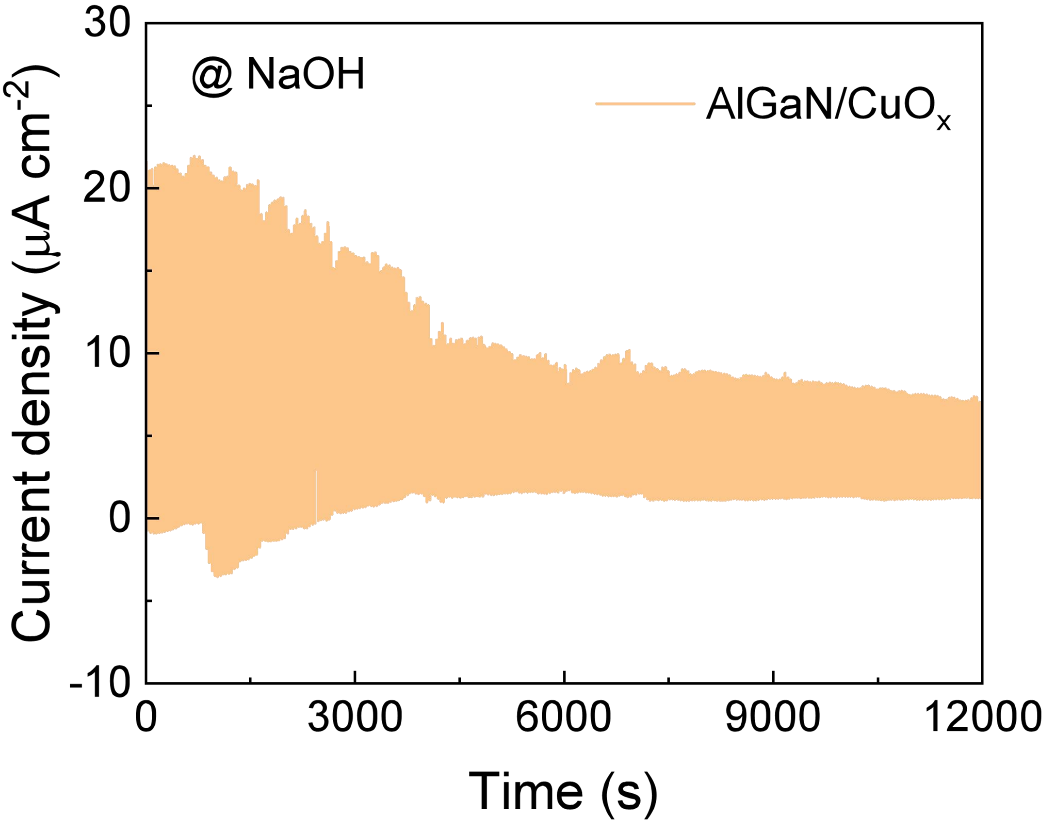

Supplementary Figure 18. Stability test under NaOH condition. 12,000-second I-t test of AlGaN/CuO_x PEC PD in alkaline NaOH electrolyte solution (0.1 M) at 255 nm light irradiation (1 mW cm^{-2}). The photoresponse performance drops over 50% within initial 12,000 s.

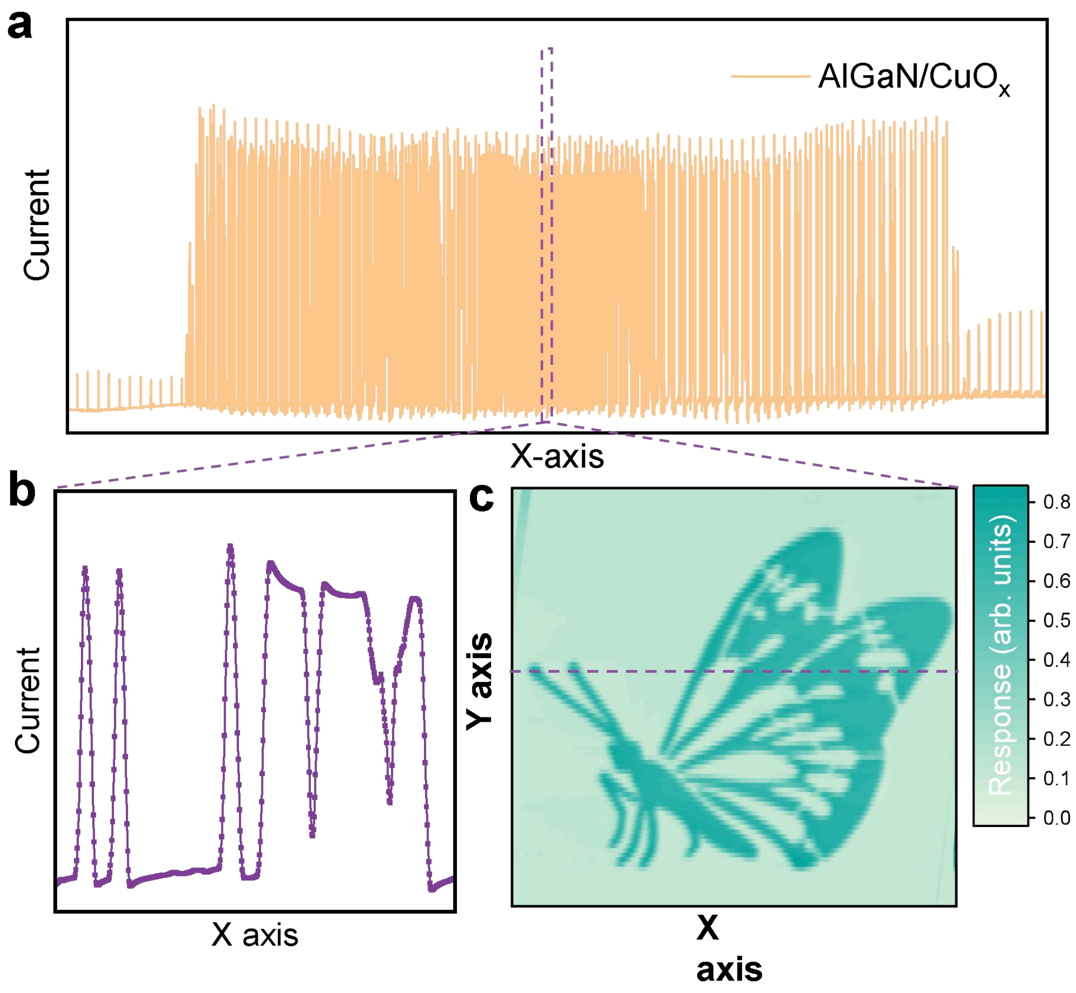

Supplementary Figure 19. Imaging with the AlGaN/CuO$_x$ PEC PD. The photocurrent response curve (**a**) of the mask scanned by the AlGaN/CuO$_x$ PEC PD, where the enlarged view (**b**) of the curve within the purple rectangle corresponds to the purple line in the imaging result graph (**c**).

The operational principles and testing procedures of the optical imaging system, which are of great significance for understanding the entire experimental process, are clearly presented by the schematic diagram shown in Fig. 6a. The mask is placed between the PEC PD and the LED light source, where it undergoes a scanning operation relying on an X-Y biaxial displacement platform. The scanning speed, which can be set within the range from 0.01 mm/s to 1 mm/s based on the desired imaging effect, and the sample interval, which can be adjusted from 0.0001 to 0.1 s, are the key factors that determine the imaging clarity. The hollow parts of the mask pattern, through which the ultraviolet light emitted by the LED can pass, serve as the main source of the photocurrent. In order to eliminate the interference of the divergent light from the LED on the quality of imaging, PVC tape with excellent light-shielding properties is used to seal the parts other than the PEC PD device (whose area is approximately 0.01 cm^2). During the testing process, the PEC PD collects current data in real-time, and these data are processed by the algorithms of MATLAB software and output in the form of a heatmap, so that the final imaging results can be obtained. The "butterfly" pattern was designed and plotted by X. Wang. The mask was fabricated by laser engraving. The elements of the image have never been used otherwhere.

Section 2. Supplementary Note

Supplementary Note 1 | Carrier transport of high-amount CuO_x decorated AlGaN nanowires at different wavelength light

When p-CuO_x/n-AlGaN nanowires are irradiated by high-energy λ_1 light, photo-induced electron-hole pairs are generated within both p-CuO_x and n-AlGaN segments. Under the driving of built-in electric field of AlGaN/CuO_x, the generated electrons and holes in AlGaN drift to si substrate and to CuO_x capping layer, respectively, resulting a positive photocurrent. At the contact interface between p-CuO_x and the electrolyte, the energy band bends downward, allowing holes to migrate toward the working electrode, resulting in small a negative current. However, the primary effect of the built-in electric field in p-CuO_x/n-AlGaN raises the n-AlGaN valence band bending potential higher than at the p-CuO_x/electrolyte contact surface, making it easier for photogenerated holes to migrate across the potential barrier to the p-CuO_x/electrolyte interface for the oxygen evolution reaction (OER); Thereout, due to the photocurrent induced by pn junction prevailing over the p-GaN/electrolyte, the total photocurrent shown by the AlGaN/CuO_x PD at λ_1 light is a positive photocurrent. When p-CuO_x/n-AlGaN nanowires are irradiated by lower-energy λ_2 light, the electron-hole pairs only can be generated within p-CuO_x segments and massive electrons transfer to electrolyte. The generated holes in p-CuO_x segments accumulate near the p-CuO_x/n-AlGaN interface, then reduce the intensity of built-in electric field and lower the hole potential barrier in valence band, which enhances the probability for the hole diffusion into the AlGaN segment. Ultimately, the high-level deposited p-CuO_x/n-AlGaN PD generats a negative photocurrent signal under low-energy λ_2 light illumination. The polarity switching on photocurrent, depending on the light wavelength, usually occurs in the pn-junction photoelectrode based PEC PD and has been reported elsewhere[1,4].

Supplementary References

1. Wang, D. *et al.* Bidirectional photocurrent in p–n heterojunction nanowires. *Nature Electronics* **4**, 645 (2021).
2. Fang, S. *et al.* Light-Induced Bipolar Photoresponse with Amplified Photocurrents in an Electrolyte-Assisted Bipolar p–n Junction. *Advanced Materials* **35**, 2300911 (2023).
3. Kang, Y. *et al.* Achieving Record-High Photoelectrochemical Photoresponse Characteristics by Employing Co_3O_4 Nanoclusters as Hole Charging Layer for Underwater Optical Communication. *ACS Nano* **17**, 3901 (2023).
4. He, C. *et al.* α-Ga2O3 Nanorod Array–Cu2O Microsphere p–n Junctions for Self-Powered Spectrum-Distinguishable Photodetectors. *ACS Applied Nano Materials* **2**, 4095, (2019).
